# Determination of the evolutionary pressure on *Camellia oleifera* on Hainan Island using the complete chloroplast genome sequence

**DOI:** 10.7717/peerj.7210

**Published:** 2019-06-26

**Authors:** Wan Zhang, Yunlin Zhao, Guiyan Yang, Jiao Peng, Shuwen Chen, Zhenggang Xu

**Affiliations:** 1 Hunan Research Center of Engineering Technology for Utilization of Environmental and Resources Plant, Central South University of Forestry and Technology, Changsha, Hunan, China; 2 College of Forestry, Northwest A & F University, Yangling, China; 3 Hunan Urban and Rural Ecological Planning and Restoration Engineering Research Center, Hunan City University, Yiyang, Hunan, China

**Keywords:** *Camellia oleifera*, Island plant, Chloroplast genome, Repeat analysis, Codon usage, SSR, Evolution pressure

## Abstract

*Camellia oleifera* is one of the four largest woody edible oil plants in the world with high ecological and medicinal values. Due to frequent interspecific hybridization, it was difficult to study its genetics and evolutionary history. This study used *C. oleifera* that was collected on Hainan Island to conduct our research. The unique island environment makes the quality of tea oil higher than that of other species grown in the mainland. Moreover, a long-term geographic isolation might affect gene structure. In order to better understand the molecular biology of this species, protect excellent germplasm resources, and promote the population genetics and phylogenetic studies of *Camellia* plants, high-throughput sequencing technology was used to obtain the chloroplast genome sequence of Hainan *C. oleifera*. The results showed that the whole chloroplast genome of *C. oleifera* in Hainan was 156,995 bp in length, with a typical quadripartite structure of a large single copy (LSC) region of 86,648 bp, a small single copy (SSC) region of 18,297 bp, and a pair of inverted repeats (IRs) of 26,025 bp. The whole genome encoded a total of 141 genes (115 different genes), including 88 protein-coding genes, 45 tRNA genes, and eight rRNA genes. Among these genes, nine genes contained one intron, two genes contained two introns, and four overlapping genes were also detected. The total GC content of Hainan *C. oleifera*’s chloroplast genome was 37.29%. The chloroplast genome structure characteristics of Hainan *C. oleifera* were compared with mainland *C. oleifera* and those of the other eight closely related Theaceae species; it was found that the contractions and expansions of the IR/LSC and IR/SSC regions affected the length of chloroplast genome. The chloroplast genome sequences of these Theaceae species were highly similar. A comparative analysis indicated that the Theaceae species were conserved in structure and evolution. A total of 51 simple sequence repeat (SSR) loci were detected in the chloroplast genome of Hainan *C. oleifera*, and all *Camellia* plants did not have pentanucleotide repeats, which could be used as a good marker in phylogenetic studies. We also detected seven long repeats, the base composition of all repeats was biased toward A/T, which was consistent with the codon bias. It was found that Hainan *C. oleifera* had a similar evolutionary relationship with *C. crapnelliana*, through the use of codons and phylogenetic analysis. This study can provide an effective genomic resource for the evolutionary history of Theaceae family.

## Introduction

The chloroplast genome, also known as chloroplast DNA, is often abbreviated as cpDNA. It shows the typical quadripartite structure generally consisting of four parts with a large single copy (LSC), a small single copy (SSC), and two inverted repeats (IRs) (Jansen et al., 2005; Palmer, 1991). The two IR regions are in the same sequence, but in the opposite directions. In general, 400–1,600 chloroplast genome copies are contained in plant cells (leaves) (Yang et al., 2010). The variation of chloroplast genome size is mainly affected by the length of the IR regions. For example, in the evolutionary process of the *Geranium* chloroplast genome, IR regions increased by 76 kb (Palmer, 1985). The IR regions of gymnosperms, such as Japanese black pine, which was only 495 bp in length (Tsudzuki et al., 1992), whereas the IR regions of legumes, such as *Medicago*, disappeared completely (Saski et al., 2005). This polymorphism of chloroplast genome has important research significance in phylogenetic and population genetics.

The chloroplast genome sequence has been successfully applied to the taxonomic and phylogenetic studies of many species. And the molecular evolution rate of the coding and non-coding regions are significantly different (Olmstead & Palmer, 1994). However, cpDNA is highly conserved (Wolfe, Li & Sharp, 1987), shows maternal inheritance (Corriveau & Coleman, 1988), and does not undergo genetic recombination. The control of chloroplast gene expression is affected by environmental factors and its developmental program, operating at several steps, including transcription, post-transcription, translation, and post-translation. The chloroplast gene expression is largely controlled at the post-transcriptional level (Sugita & Sugiura, 1996). Therefore, the plant chloroplast genome has significant advantages in revealing the relationship of species, and can provide a large amount of important data on chloroplast genetic transformation.

*Camellia oleifera* belongs to the genus *Camellia* of the family Theaceae. It is mainly distributed in the subtropical mountains of China (Zhou et al., 2013; Zhuang, 2008). A forest of *C. oleifera* has a good ecological value in maintaining soil and water and in regulating climate. In addition, its tea oil has a high medicinal value (He et al., 2007). In this study, we chose the *Camellia* species with a unique living environment (on Hainan Island), which is different from the other *Camellia* distribution provinces in China. The unique island climatic conditions have produced excellent and unique *C. oleifera* resources (Zheng et al., 2016). However, so far, the background in the genetics and genomics research of *Camellia* is rather weak. Due to frequent interspecific hybridization and polyploidization, the classification and phylogenetic studies of *C. oleifera* are quite difficult (Huang et al., 2014). Moreover, the *C. oleifera* on Hainan Island is an independent population, has a certain geographic isolation, with a relatively large genetic variation of its population. In the long run, this will gradually lead to the loss of the resources of germ-plasm. Therefore, this study will analyze the chloroplast genomic sequence of the Hainan *C. oleifera* to understand its similarities and differences with the other *Camellia* species in the process of natural selection, and identify its taxonomic groups, thereby providing valuable genomic resources for molecular breeding and phylogenetic research.

## Materials and Methods

### DNA extraction, sequencing, and annotation

Fresh leaves of *C. oleifera* were obtained by germinating the seeds that were collected from Chengmai, Hainan Province (110.00°E, 19.75°N) in China and the seeds were collected in areas that were not privately owned or protected in any way and no specific permits were required for this study. The treatment of plants and DNA extraction refer to the method of Zhang et al. (2017) and more experiment details could be got from our announcement.

The genome can be assembled directly by using overlap between sequenced reads; a total of 2.83G raw data was obtained, including 2.09G of clean data. The quality of the sequencing data of the samples was visually evaluated by the software Fastqc v 0.10.0 (http://www.bioinformatics.babraham.ac.uk/projects/fastqc/). After the Illumina PCR adapter reads and low-quality reads were filtered from the paired-end and mate-pair library in the quality control step, all good-quality paired reads were assembled to contigs by using SOAPdenovo2 (Luo et al., 2012). The assembled contigs were joined into multiple scaffolds using SSPACE (Boetzer et al., 2011) to obtain the whole-genome sequence.

The programs DOGMA (Wyman, Jansen & Boore, 2004) and cpGAVAS (Liu et al., 2012) were used for the functional annotation and gene prediction of the whole chloroplast genome, respectively. Subsequently, the start and stop codons were adjusted manually by means of the Sequin tool to get the final open reading frame (ORF). The GeneMarkS (version 4.17) (http://topaz.gatech.edu/) program was used to retrieve the related coding genes. The predicted protein sequences were aligned using the National Center for Biotechnology Information (NCBI) Conserved Domain Database CDD (Marchlerbauer et al., 2015) to predict the Clusters of Orthologous Group of Proteins function of the genes and make the classification statistics (Yao et al., 2015). The circular chloroplast genome map of Hainan *C. oleifera* was generated with the OGDRAW (Lohse, Drechsel & Bock, 2007) software. The complete chloroplast genome sequence of Hainan *C. oleifera* has been submitted to the NCBI with GenBank accession number MF541730.

### Comparative analysis of chloroplast genomes

The number of protein-coding genes, the GC content and the length of each region in the Hainan *C. oleifera* chloroplast genome and other nine Theaceae plants were compered by manually. A multiple sequence alignment of the 10 Theaceae chloroplast genomes was performed using mVISTA (Frazer et al., 2004; Mayor et al., 2000), with the annotated Hainan *C. oleifera* chloroplast genome sequence as a reference. The following nine species (with their GenBank accession numbers given in parentheses) are members of the genus *Camellia*: *C. oleifera* (JQ975031), *C. luteoflora* (KY626042), *C. grandibracteata* (NC_024659), *C. sinensis* (KC143082), *C. leptophylla* (NC_024660), *C. pubicosta* (NC_024662), *C. crapnelliana* (KF753632), *C. huana* (KY626040), and *C. danzaiensis* (NC_022460), which were downloaded from the GenBank database of NCBI (https://www.ncbi.nlm.nih.gov/pubmed). The visual comparisons of the IR/LSC and IR/SSC border regions of chloroplast genome from nine different species closely related to Hainan *C. oleifera* were conducted using the drawing software Visio 2013.

### DNA barcode compared of Hainan *C. oleifera* and Mainland species

We selected the important DNA barcode sequences *matK* and *trnH-psbA* to compare Hainan *C. oleifera* and Mainland *C. oleifera*. The following objects: *C. oleifera-matK* (KP094074, KP094075, KR530501, KX216462) and *C. oleifera-trnH-psbA* (KX121760, KR533766, KP095297, GQ435325, GQ435326, GQ487355, KP095298) were involved. All registration numbers can be found in NCBI. The nucleotide sequence information of some *C. oleifera* was searched by NCBI database blast, and compared with the same nucleotide position of Hainan *C. oleifera* by the software MEGA 7.0.

### SSRs characterization and long repeat sequences

The simple sequence repeats (SSRs) are also known as the short tandem repeats or microsatellites. Because of the presence of different nucleotides in the repeat units and different number of repeats, a high degree of variability in SSR length was caused. The microsatellite (di-, tri-, tetra-, penta-, and hexa-nucleotide repeats) assays were performed by using the software SSRHunter 1.3 (Li & Wan, 2005) with a minimum number of four repeat units for di- and tri-nucleotides and three for tetra-, penta-, and hexa-nucleotides.

The REPuter (Kurtz et al., 2001) software was used to predict the chloroplast genome long repeat sequences, which were divided into four categories, forward match, reverse match, palindromic match and complementary match, according to their comparison. In this paper, we examined the long repeat sequences with a minimum repeat length of 30 bp and a maximum of 60 bp.

### Indices of codon usage

The relative synonymous codon usage (RSCU) (Sharp & Li, 1987) of Hainan *C. oleifera* chloroplast genome was calculated using DAMBE6 (Xia, 2017) based on the sequence of protein-coding genes in the chloroplast genome. The obtained RSCU values were statistically analyzed by SigmaPlot 10.0 and plotted into a histogram. The distribution of codon usage for the 10 Theaceae species is shown in the form of a heatmap, which was constructed using HemI 1.0 (Deng et al., 2014), according to the RSCU value. As an intuitive representation, it can graphically display the matrix data by describing each value in different color.

### Sequence phylogenetic analysis

Evolutionary trees that indicate the evolutionary relationships among species which are thought to have a common ancestor are referred to as phylogenetic trees. The maximum likelihood method was used to determine the phylogenetic relationships of the Hainan *C. oleifera* clade with the clades of 21 other Theaceae species using MEGA 7.0 (Kumar, Stecher & Tamura, 2016) and an online software Interactive Tree of Life (iTOL) (Letunic & Bork, 2016). The GenBank databases was used to download the amino acid sequences 21 *Camellia* species, including *C. oleifera* (JQ975031), *C. huana* (KY626040), *C. impressinervis* (KF156835), *C. pitardii* (KF156837), *C. danzaiensis* (KF156834), *C. grandibracteata* (KJ806274), *C. leptophylla* (KJ806275), *C. sinensis var. sinensis* (KJ806281), *C. sinensis var. dehungensis* (KJ806279), *C. crapnelliana* (KF753632), *C. taliensis* voucher HKAS:S.X.Yang3157 (KF156839), *C. taliensis* voucher HKAS:S.X.Yang3158 (KF156836), *C. reticulata* (KJ806278), *C. sinensis var. pubilimba* (KJ806280), *C. pubicosta* (KJ806277), *C. sinensis* (KC143082), *C. sinensis* cultivar Longjing 43 (KF562708), *C. cuspidata* voucher HKAS: S.X.Yang3159 (KF156833), *C. yunnanensis* voucher HKAS:S.X.Yang1090 (KF156838), *C. petelotii* (KJ806276), *C. luteoflora* voucher CLUTE20161220 (KY626042). The model for phylogenetic assessment was based on the Newick tree file generated by MEGA 7.0. The tree was then imported to the iTOL online system. The bootstrap was quickly calculated with the iTOL’s in-built system, followed by the construction of the final circular phylogenetic tree. The same color-coded species in the picture represented similar phylogenetic relationships. We constructed phylogenetic trees using the following datasets due to the different molecular evolution rates of different cp genomic regions: (1) Complete chloroplast genome sequence; (2) Protein coding sequence; (3) LSC region; (4) SSC region. On the other hand, barcode labeling has been successfully applied to the study of phylogenetic relationships, and we have also compared the base sequences of DNA barcode (*rbcL*, *matK*, and *trnH-psbA*) of Hainan *C. oleifera* chloroplast genome and other 21 Theaceae plants. These three sites are commonly used research objects in DNA barcodes and are important molecular tools for identifying organisms (CBOL Plant Working Group, 2009; Yang et al., 2016).

## Results and Analysis

### Chloroplast genome characteristic of Hainan *C. oleifera*

**Figure 1:**
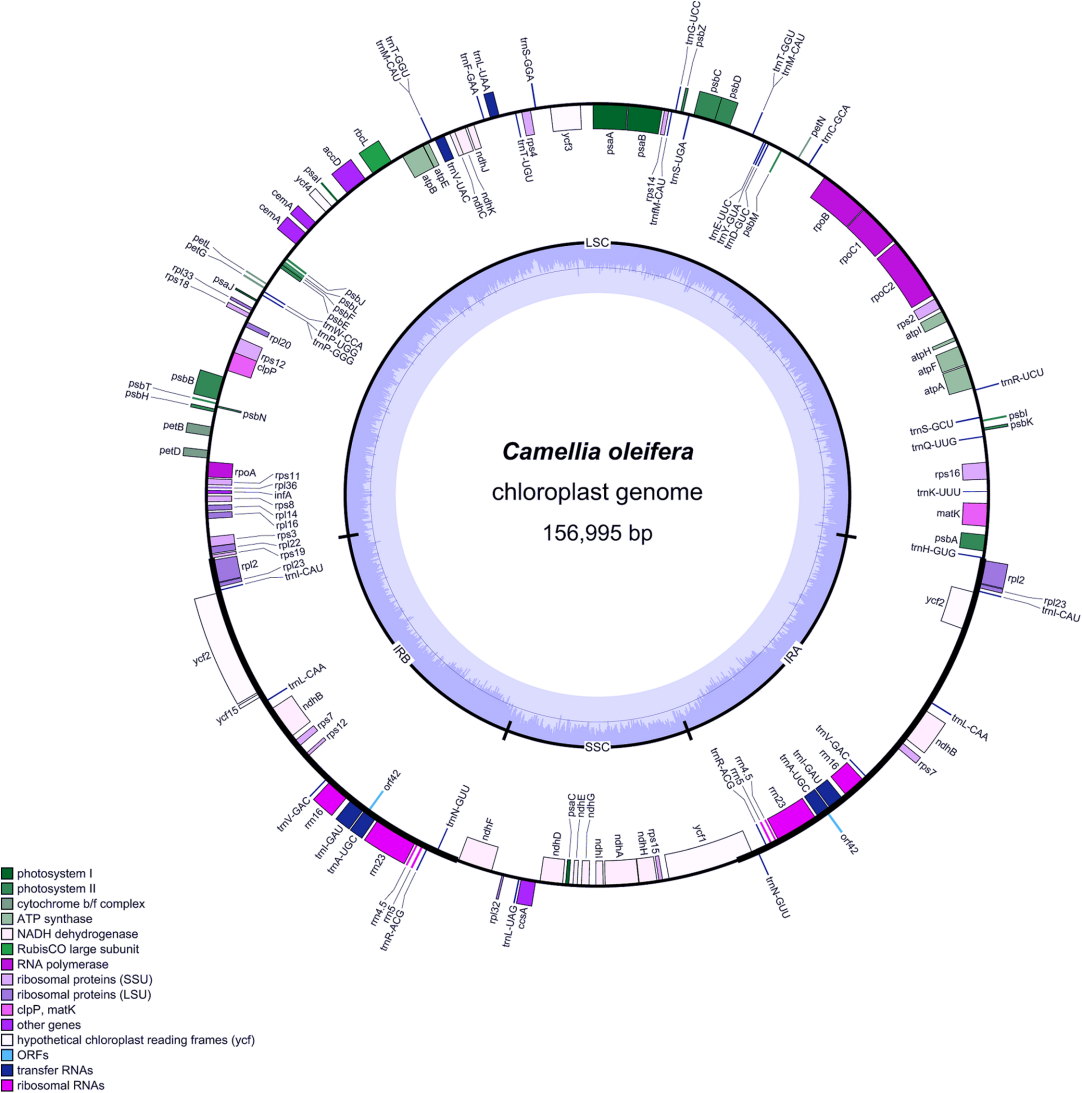
Gene map of the Hainan *C. oleifera* chloroplast genome. The outermost colored blocks represent the physical location of different genes on the chloroplast genome, the inner circle is the physical location of the LSC, SSC, and IR regions on the genome and the different colors represent genes of different functional classes. Genes distributed outside the circle are transcribed counterclockwise, whereas genes inside are transcribed clockwise. The dark gray in the inner circle represents GC content, and the light gray represents AT content.

Like the majority of land plants, the whole chloroplast genome of Hainan *C. oleifera* showed a typical quadripartite genome organization with a size of 156,995 bp, including a LSC region of 86,648 bp and a SSC region of 18,297 bp, which were separated by two IR (IRa and IRb) regions of 26,025 bp (Fig. 1).

The chloroplast genome of Hainan *C. oleifera* was found to contain 141 predicted functional genes, including 88 protein-coding genes, 45 tRNA genes and eight rRNA genes, which were classified according to their function. Among the observed genes, 81 protein-coding genes, 30 tRNA genes, and four rRNA genes were unique. Among the protein-coding genes, 74 were single-copy genes and seven (*ndhB*, *rps12*, *rps7*, *rpl2*, *rpl23*, *ycf2*, and *orf42*) were duplicates. Among the tRNA genes, 19 were unique, and 11 (*trnA-UGC, trnI-CAU*, *trnI-GAU*, *trnL-CAA*, *trnL-UAA*, *trnM-CAU*, *trnN-GUU*, *trnR-ACG*, *ttrnT-GGU*, *trnV-GAC*, and *trnV-UAC*) were duplicates. The four rRNA genes were completely duplicated (Table 1). In addition, four overlapping genes (*psbD*-*psbC*, *ndhK*-*ndhC*, *trnM-CAU*-*trnT-GGU*, and *trnP-GGG*-*trnP-UGG*) were detected. Among these genes, nine genes (*atpF*, *rpoC1*, *rpl2*, *ndhA*, *ndhB*, *ycf1*, and *trnI-GAU*) contained a single intron, of which *rpl2* and *ndhB* were identified as repetitive genes. Two genes (*ycf3* and *clpP*) contained two introns. These two genes were similar to those in the Orpheus flower *Haberlea rhodopensis* and *Broussonetia papyrifera* (Ivanova et al., 2017; Xu et al., 2018). Unlike most angiosperm chloroplast genomes (Jin et al., 2014; Kong & Yang, 2015; Wang et al., 2016), among the 11 genes, four (*atpF*, *rpoC1*, *ycf3*, *clpP*) of which were located in the LSC region, two (*ndhA* and *trnI-GAU*) in the SSC region, other four (*rpl2*×2, *ndhB*×2) in the IR region, and one gene (*ycf1*) was a trans-spliced gene with its start codon located in the SSC region, stop codon in the IRb region, and possibly missing 3′ end. Additionally, the *ndhA* gene contained the largest intron (1,093 bp), and the *ycf1* gene had the smallest one (27 bp) (Table S1).

**Table 1:** Genes present in the Hainan *C. oleifera* chloroplast genome.

Group of genes	Gene names
Photosystem I	*psaA*, *psaB*, *psaC*, *psaI*, *psaJ*
Photosystem II	*psbA*, *psbB*, *psbC*, *psbD*, *psbE*, *psbF*, *psbH*, *psbI*, *psbJ*, *psbK*, *psbL*, *psbM*, *psbN*, *psbT*, *psbZ*
Cytochrome b/f complex	*petA*, *petB*, *petD*, *petG*, *petL*, *petN*
ATP synthase	*atpA*, *atpB*, *atpE*, atpF*, *atpH*, *atpI*
NADH dehydrogenase	ndhA*, *ndhB*(×2)*, *ndhC*, *ndhD*, *ndhE*, *ndhF*, *ndhG*, *ndhH*, *ndhI*, *ndhJ*, *ndhK*
RubisCO large subunit	*rbcL*
RNA polymerase	*rpoA*, *rpoB*, rpoC1*, *rpoC2*
Ribosomal proteins (SSU)	*rps11*, *rps12*(×2), *rps14*, *rps15*, *rps16*, *rps18*, *rps19*, *rps2*, *rps3*, *rps4*, *rps7*(×2), *rps8*
Ribosomal proteins (LSU)	*rpl14*, *rpl16*, *rpl2*(×2)*, *rpl20*, *rpl22*, *rpl23*(×2), *rpl32*, *rpl33*, *rpl36*
Proteins of unknown function	ycf1*, *ycf15*, *ycf2*(×2), ycf3**, *ycf4*
Transfer RNAs	*trnA-UGC*(×4)*, trnC-GCA*, *trnD-GUC*, *trnE-UUC*, *trnF-GAA*, *trnfM-CAU*, *trnG-UCC*, *trnH-GUG*, *trnI-CAU*(×2), *trnI-GAU*(×4), *trnK-UUU*, *trnL-CAA*(×2), *trnL-UAA*(×2), *trnL-UAG*, *trnM-CAU*(×2), *trnN-GUU*(×2), *trnP-GGG*, *trnP-UGG*, *trnQ-UUG*, *trnR-ACG*(×2), *trnR-UCU*, *trnS-GCU*, *trnS-GGA*, *trnS-UGA*, *trnT-GGU*(×2), *trnT-UGU*, *trnV-GAC*(×2), *trnV-UAC*(×2), *trnW-CCA*, *trnY-GUA*
Ribosomal RNAs	*rrn16*(×2), *rrn23*(×2), *rrn4.5*(×2), *rrn5*(×2)
Other genes	*infA*, *matK*, clpP**, *accD*, *ccsA*, *cemA*, *orf42*(×2)

**Notes:**

* One or two asterisks after genes indicate that gene contains one or two introns, respectively.

The numbers in parentheses indicate the copy number of the gene.

### Comparison with other Theaceae chloroplast genomes

The chloroplast genomic characteristics of Hainan *C. oleifera* were compared with nine other Theaceae species (Table 2). Similar to the reported results of the Theaceae chloroplast genome (Shi et al., 2013; Yang et al., 2013), the whole-genome length of Hainan *C. oleifera* was not much different from that of the other species. This change in sequence length depends mainly on the difference in the length of the LSC region (Li et al., 2015). The GC contents of the 10 species of *Camellia* were 37.29–37.34%, which were unevenly distributed in the whole genomes, with IRs exhibiting the highest GC content (42.94–43.01%), followed by the LSC (35.29–35.36%) and SSC regions (30.52–30.63%). Among them, the GC content in each area of Hainan *C. oleifera* and *C. oleifera* was the closest. As an important marker of molecular evolution, the IR region has a high GC content, which makes the sequence stable and highly conserved. In general, the protein-coding genes of two *C. oleifera* were the closest, although the total number of genes in Hainan *C. oleifera* was much larger than that of mainland *C. oleifera*, which has the largest number of 141 genes. This phenomenon may be caused by gene recombination and may affect its evolution.

**Table 2:** The basic composition of the Hainan *C. oleifera* chloroplast genome and other nine Theaceae plants.

	Total sequence length (bp)/GC content	LSC length/GC content	SSC length/GC content	IR length/GC content	Number of genes	Protein-coding genes
*C. oleifera* Hainan	156,995/37.29%	86,648/35.29%	18,297/30.55%	26,025/42.98%	141	88
*C. oleifera*	156,971/37.31%	86,515/35.30%	18,288/30.54%	26,084/42.98%	133	87
*C. luteoflora*	157,166/37.30%	86,719/35.32%	18,293/30.59%	26,077/42.96%	133	91
*C. grandibracteata*	157,127/37.29%	86,656/35.32%	18,285/30.52%	26,093/42.94%	127	91
*C. sinensis*	157,103/37.31%	86,645/35.34%	18,276/30.54%	26,091/42.95%	135	91
*C. leptophylla*	157,102/37.30%	86,647/35.32%	18,275/30.58%	26,090/42.95%	126	91
*C. pubicosta*	157,076/37.30%	86,649/35.33%	18,279/30.54%	26,074/42.95%	127	91
*C. crapnelliana*	156,997/37.30%	86,655/35.30%	18,406/30.60%	25,968/43.01%	136	91
*C. huana*	156,903/37.32%	86,568/35.34%	18,203/30.63%	26,066/42.96%	133	92
*C. danzaiensis*	156,576/37.34%	86,204/35.36%	18,259/30.59%	26,056/42.98%	135	92

**Figure 2:**
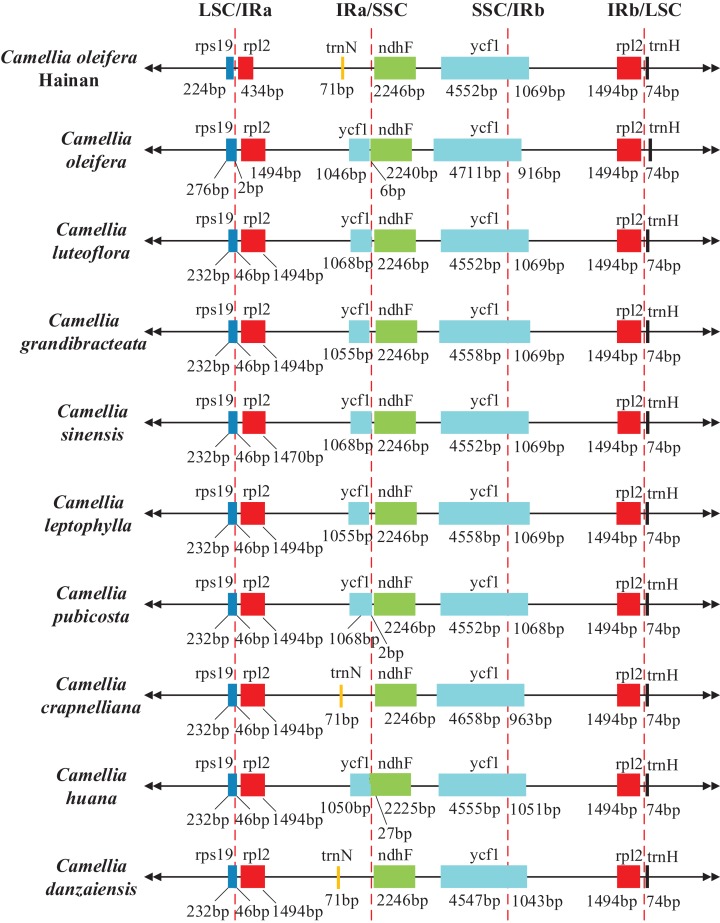
Comparison of the border positions of LSC, SSC, and IRs regions in chloroplast genome sequences of 10 Theaceae species.

In the present paper, the IR/LSC and IR/SSC regions of the Hainan *C. oleifera* chloroplast genome were compared with the corresponding regions of the nine closely related Theaceae species (Fig. 2). As can be seen from the other *Camellia* chloroplast genome studies (Huang et al., 2014), the length of the chloroplast genome was variable, mainly due to the expansions and contractions of the border regions. Consistently, in all of the comparative chloroplast genomes, the IRb/SSC regions were located in the coding sequences of the *ycf1* gene. The *rpl2* (1,494 bp) and *trnH* (74 bp) genes were located on both sides of the IRb/LSC regions and were 106 and 2 bp away from the borders, respectively. However, the *rps19* gene was located in the LSC region because of the expansion of the Hainan *C. oleifera* LSC region border, which was eight bp apart from the IRa/LSC region. Similarly, the *rps19* gene from the mainland *C. oleifera* was also more biased towards the LSC region, whereas in the other eight *Camellia* species, with 232 bp occupying the LSC region and 46 bp occupying the IRa region. Like most plants, the *ndhF* gene involved in photosynthesis was located in the SSC region (Shen et al., 2017) and was 2,246 bp in length in all *Camellia* species, except in *C. huana* and *C. oleifera*. It should be emphasized that the *ycf1* gene in the IRa regions was not observed in Hainan *C. oleifera*, *C. crapnelliana*, and *C. danzaiensis* because of the contraction of the IRa region border, which was replaced by the *trnN* gene. By comparing the border regions of the chloroplast genomes of 10 Theaceae species, it can be summarized as two different types and main feathered by gene structure of IRa/SSC. The *trnN*-*ndhF* genes located in above region of Hainan *C. oleifera*, *C. crapnelliana* and *C. danzaniensis*, while other species were *ycf1*-*ndhF*.

**Figure 3:**
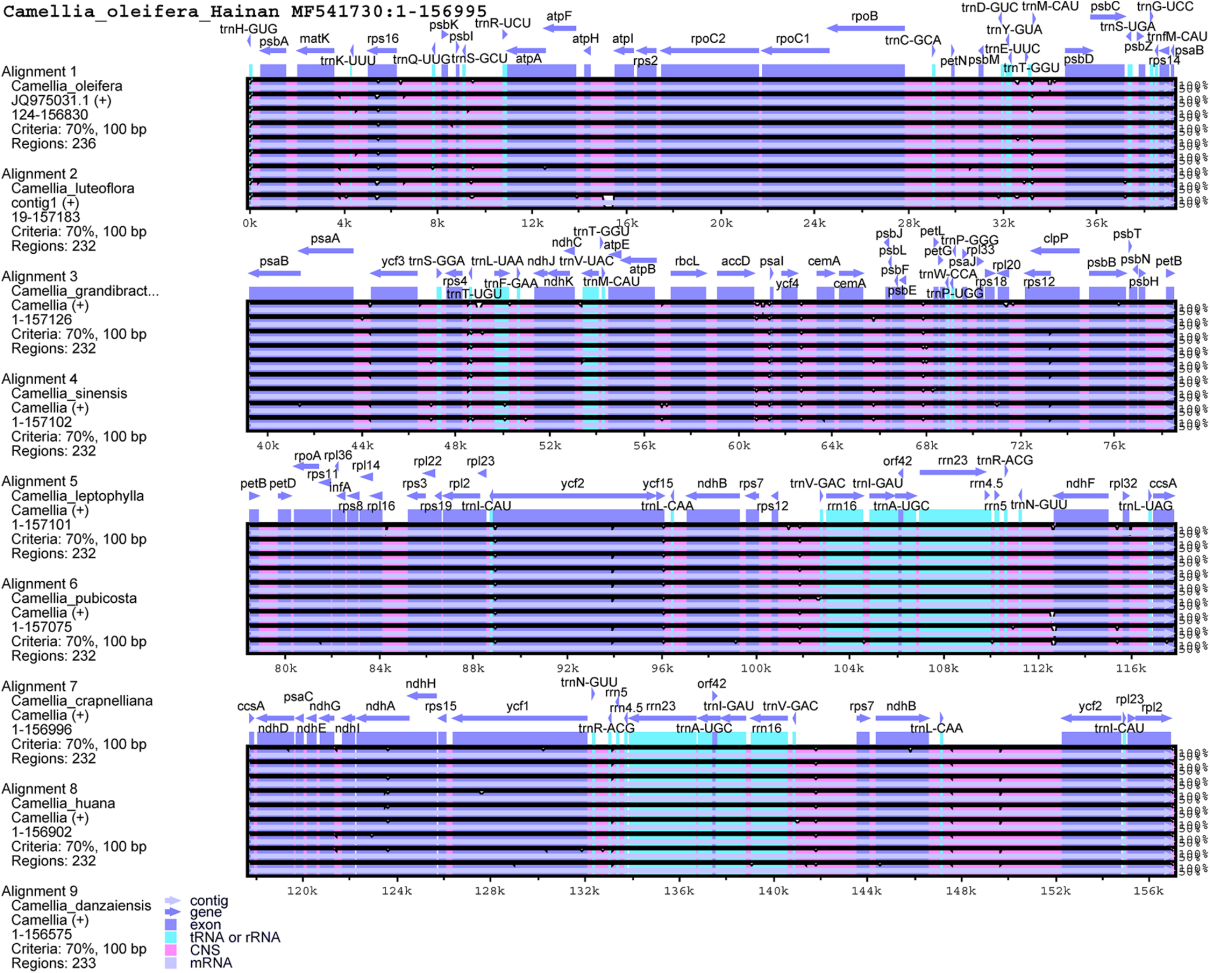
Visualization alignments of chloroplast genome sequences among 10 Theaceae species with Hainan *C. oleifera* chloroplast genome as a reference. The abscissa represents the position coordinates of the chloroplast genome of Hainan *C. oleifera*, and the ordinate represents the sequence similarity of the sample genome to the reference genome. Arrows indicate the annotated gene and its transcriptional direction, blue for the protein coding sequence (exons), green for tRNA or rRNA, and red for the conserved non-coding sequence (CNS).

A multiple sequence alignment of the chloroplast genomes of the 10 Theaceae species was presented in Fig. 3. The result showed a high degree of similarity in the chloroplast genome sequences, suggesting that the chloroplast genome in the Theaceae species was evolutionarily conserved, despite of the discovery of several different regions in it, while the conservative of Theaceae plants is relatively rare in all angiosperms, which also makes the study of its evolution a certain pressure. Like other plants, the non-coding regions showed higher sequence diversity than the coding regions, whereas the IRs regions were more conserved than the single-copy regions (Huang et al., 2014; Ivanova et al., 2017; Yao et al., 2015). Among them, the *rps16*, *clpP*, *ndhA*, *ycf2*, and *ycf1* genes showed high sequence divergence in the coding regions that makes such genes reliable markers for phylogenetic analysis. In addition, the non-coding regions showing a high degree of divergence included *trnK-UUU-rps16*, *ycf3-trnS-GGA*, *trnL-UAA-trnF-GAA*, *accD-psaI*, *ycf4-cemA*, *psbE-petL*, *rps12-trnV-GAC*, *trnL-CAA-ycf2*, and *trnV-GAC-rps7*.

### Alignment of the *matK* gene and *trnH-psbA* IGR with other *Camellia oleifera*

**Figure 4:**
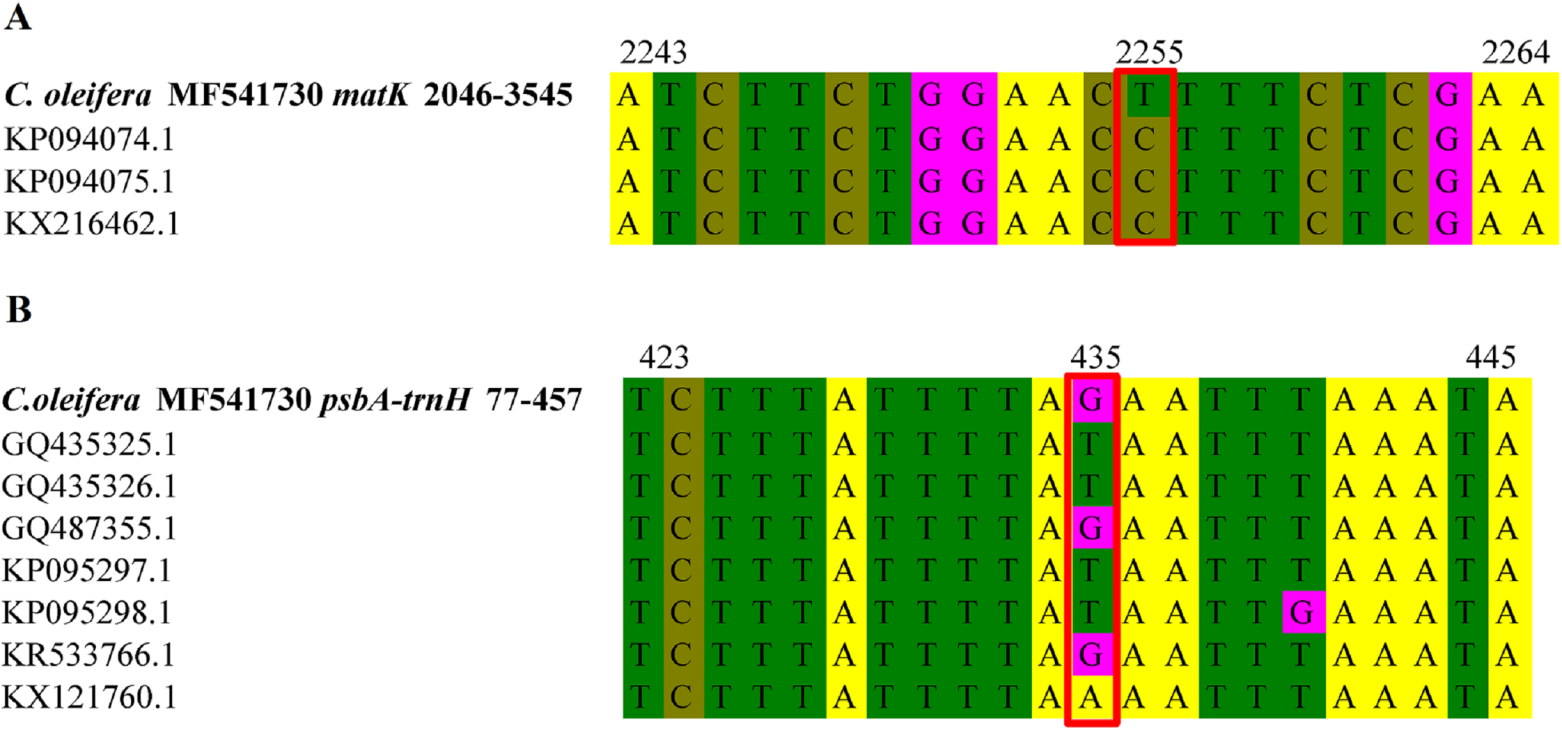
Base sequence alignment of *matK* gene and *trnH-psbA* intergenic region between different *C. oleifera*. (A) Partial nucleotide sequence of *matK* gene; (B) partial nucleotide sequence of *trnH-psbA* intergenic region.

We compared Hainan *C. oleifera* with other mainland *C. oleifera* published in the NCBI database. The base sequences of the *matK* gene and *trnH-psbA* noncoding spacer were shown in Fig. 4. We selected some bases with differences, among which *matK* coding genes were compared. Although the protein base sequence is conservative, the results showed that there was a site change in Hainan *C. oleifera*, that is, at the position of 2,255 bp, all other three mainlands *C. oleifera* were base C, and object species was T. Similarly, in the comparison of *trnH-psbA*, it also changed at 435 bp, but it was the same as GQ487355 and KR533766, which were base G, and the other plants were A or T. This substitution of base pairs may cause changes in gene structure, leading to genetic mutations, which may be related to the living environment of Hainan *C. oleifera*.

### SSR and long repeat sequences distribution

The SSRs are repetitive sequences of up to several dozen nucleotides consisting of one to six nucleotide repeat units, which are short in length and widely distributed in eukaryotic chloroplast genomes (Chen et al., 2006; Goldstein & Schlötterer, 1999). Owing to the presence of different nucleotides in the repeat units and different number of repeats, of which the most common is the dinucleotide repeat type, a high degree of variability is generated in SSR length. Therefore, we detected the SSRs distribution of the Hainan *C. oleifera* chloroplast genome by selecting the perfectly matched sequences, starting from the dinucleotide repeats. The statistical results were shown in Table 3. We detected a total of 51 SSR loci, all having a base composition biased toward A/T. Of these 51 SSRs, 28 were completely composed of A/T; this was consistent with previous studies (Cauzsantos et al., 2017; Shen et al., 2017; Yi & Kim, 2012). The distribution of SSRs in the chloroplast genome was heterogeneous, with up to 28 SSRs in the LSC region, five in the SSC region, and 18 in the IR region. Among all SSR loci, 23 were located in the 14 protein-coding genes (*matK*, *atpA*, *rpoC2*×2, *rpoC1*, *ycf3*, *cemA*, *petA*, *rpoA*, *rol22*, *rpl2*×2, *ycf2*×6, *ndhB*×2, *ndhD*×2, *ndhA*), and 26 in the intergenic and non-coding regions (*trnS-UGA*, *rrn23*×2). In terms of the SSR types, the most abundant repeat pattern was dinucleotide repeat unit (38 SSRs), with the largest type being AT/TA, accounting for 65.8% (25 SSRs). In addition, one tri- and 12 tetra-nucleotide repeats were found in the Hainan *C. oleifera* chloroplast genome. However, compared with other Theaceae species, Hainan *C. oleifera* and *C. luteoflora* were found to have no hexanucleotide repeats (Table S2). More specifically, all *Camellia* species have no pentanucleotide repeats (Huang et al., 2014). This is why these sequences were well-tagged in the studies of population phylogeny and taxonomy (Melotto-Passarin et al., 2011; Provan, Powell & Hollingsworth, 2001).

**Table 3:** Simple sequence repeats in Hainan *C. oleifera* chloroplast genome.

ID	Repeat unit	Repeat number	Length (bp)	Start	End	Region	Annotation
1	TA	4	8	2330	2337	LSC	*matK*
2	AT	4	8	4652	4659	LSC	
3	AGAT	3	12	6696	6707	LSC	
4	TC	4	8	9184	9191	LSC	
5	GTCT	3	12	11990	12001	LSC	*atpA*
6	AT	4	8	20077	20084	LSC	*rpoC2*
7	AT	5	10	20842	20851	LSC	*rpoC2*
8	AT	4	8	21871	21878	LSC	*rpoC1*
9	GA	4	8	30227	30234	LSC	
10	AG	4	8	31910	31917	LSC	
11	TCTT	3	109	34002	34110	LSC	
12	GA	4	8	37363	37370	LSC	*trnS-UGA*
13	AT	4	8	38208	38215	LSC	
14	TTTC	3	12	45247	45258	LSC	*ycf3*
15	TA	4	8	48399	48406	LSC	
16	AT	4	75	49339	49413	LSC	
17	AT	4	8	56936	56943	LSC	
18	TA	4	8	60906	60913	LSC	
19	AAAT	3	12	62713	62724	LSC	
20	TC	4	8	63432	63439	LSC	*cemA*
21	AT	4	8	64367	64374	LSC	*petA*
22	AT	4	8	65980	65987	LSC	
23	TTC	4	12	70097	70108	LSC	
24	TA	4	8	70419	70426	LSC	
25	AT	4	8	79616	79623	LSC	
26	TA	4	8	80572	80579	LSC	*rpoA*
27	AT	5	10	84341	84350	LSC	
28	AT	4	8	85931	85938	LSC	*rpl22*
29	TA	5	10	87305	87314	IRa	*rpl2*
30	GA	4	8	88915	88922	IRa	*ycf2*
31	GA	4	8	89902	89909	IRa	*ycf2*
32	TCTA	3	12	94648	94659	IRa	*ycf2*
33	TA	4	8	95494	95501	IRa	*ycf2*
34	AG	4	8	97417	97424	IRa	*ndhB*
35	TA	4	8	99589	99596	IRa	
36	CT	4	8	108654	108661	IRa	*rrn23*
37	CCCT	3	12	110067	110078	IRa	
38	AT	4	8	116288	116295	SSC	
39	GAAA	3	12	118178	118189	SSC	*ndhD*
40	AATA	3	12	118328	118339	SSC	*ndhD*
41	AAAT	3	12	121323	121334	SSC	
42	AT	4	8	123859	123866	SSC	*ndhA*
43	GAGG	3	12	133565	133576	IRb	
44	AG	4	8	134983	134990	IRb	*rrn23*
45	TA	4	8	144048	144055	IRb	
46	CT	4	8	146220	146227	IRb	*ndhB*
47	TA	4	8	148143	148150	IRb	
48	ATAG	3	12	148984	148995	IRb	
49	TC	4	8	153735	153742	IRb	*ycf2*
50	TC	4	8	154722	154729	IRb	*ycf2*
51	AT	5	10	156329	156338	IRb	*rpl2*

To further explore the evolutionary characteristics of the *Camellia* species, we compared the long-repeat sequences in the chloroplast genome of Hainan *C. oleifera* with those of the other nine species (Table 4). We identified 28 forward, reverse and palindromic repeats with lengths ranging from 30 to 60 bp in 10 Theaceae species. Of these 28 repeat sequences, the *ycf2* gene showed the presence of the largest number (60 bp in the chloroplast genome of *C. pubicosta*), and several repeats were also found in the intergenic regions. This phenomenon has also been reported in several angiosperms (Kong & Yang, 2017; Wang et al., 2016). Among them, seven long repeats ranging from 30 to 46 bp were found in the Hainan *C. oleifera* chloroplast genome. In total, six long repeats were located in the intergenic regions, and only one forward repeat was located in the protein-coding gene (*ycf2*). In the LSC regions, we observed only two pairs of palindromic repeats with lengths of 30 and 46 bp, respectively, and the remaining were located in the IR regions. Compared with the other nine species, the Hainan *C. oleifera* chloroplast genome had the same number of long-repeat sequences as *C. crapnelliana*, which had the least number of long-repeat sequences, while the most detected in *C. oleifera* (18 repeats) and the reverse match sequence was only detected in *C. oleifera*. Among the remaining seven species, eight long-repeat sequences were present in *C. luteoflora*, 12 in *C. grandibracteata*, 12 in *C. sinensis*, 10 in *C. leptophylla*, nine in *C. pubicosta*, 10 in *C. huana*, and 13 in *C. danzaiensis*.

**Table 4:** Long repeat sequences in the chloroplast genome of 10 Theaceae species.

Type	Repeat sizes (bp)										Location	Region
	*C. oleifera* Hainan	*C. oleifera*	*C. luteoflora*	*C. grandibracteata*	*C. sinensis*	*C. leptophylla*	*C. pubicosta*	*C. crapnelliana*	*C. huana*	*C. danzaiensis*		
F		56		56	56	56			56	48	*ycf2*; *ycf2*	IRa
P		56		56	56	56			56	48	*ycf2*; *ycf2*	IRa, IRb
P				48	48		60				*ycf2*; *ycf2*	IRa, IRb
F		56		48	48	56	56		56	48	*ycf2*; *ycf2*	IRb
P		48									IGS (*rpl2-trnH*; *rps19-rpl2*)	LSC, IRa
F		48									IGS (*rpl2-trnH*); *rpl2*	LSC, IRb
F		47									IGS (*rpl2-trnH*)	LSC, IRb
P	46										IGS (*petB-petD*)	LSC
P		46	46	42	42	42	42	46	46	46	*petD*; *petD*	LSC
F	42	42	41	42	42	42	42	42	42	42	IGS (*rps12-trnV*); Intron (*ndhA*)	IRa, SSC
P	42	42	41	42	42	42	42	42	42	42	IGS (*trnV-rps12*); Intron (*ndhA*)	IRb, SSC
F	38	38	38	38	38	38	38	38	38	30	*ycf2*; *ycf2*	IRa
P		38		38	38	38	38	38	38		*ycf2*; *ycf2*	IRa, IRb
F		38	38	30	30	38	38	38	38	30	*ycf2*; *ycf2*	IRb
F	38										IGS (*trnL-ycf2*)	IRb
P			38								*ycf2*; *ycf2*	IRb
P	38										*ycf2*; IGS (*trnL-ycf2*)	IRa, IRb
F										34	IGS (*rrn4.5-rrn5*)	IRa
P										34	IGS (*rrn4.5-rrn5*; *rrn5-rrn4.5*)	IRa, IRb
F										34	IGS (*rrn5-rrn4.5*)	IRb
R		32									IGS (*atpB-rbcL*)	LSC
R		31									IGS (*atpB-rbcL*)	LSC
F		31									IGS (*atpB-rbcL*)	LSC
F			34								IGS (*petA-psbJ*)	LSC
P	30	30	30	30	30	30	30	30	30	30	IGS (*psbI-trnS*; *trnS-rps4*)	LSC
R		30									IGS (*atpB-rbcL*)	LSC
F		30									IGS (*atpB-rbcL*)	LSC
P				30	30					30	*ycf2*; *ycf2*	IRa, IRb

### Characteristic of codon usage

**Figure 5:**
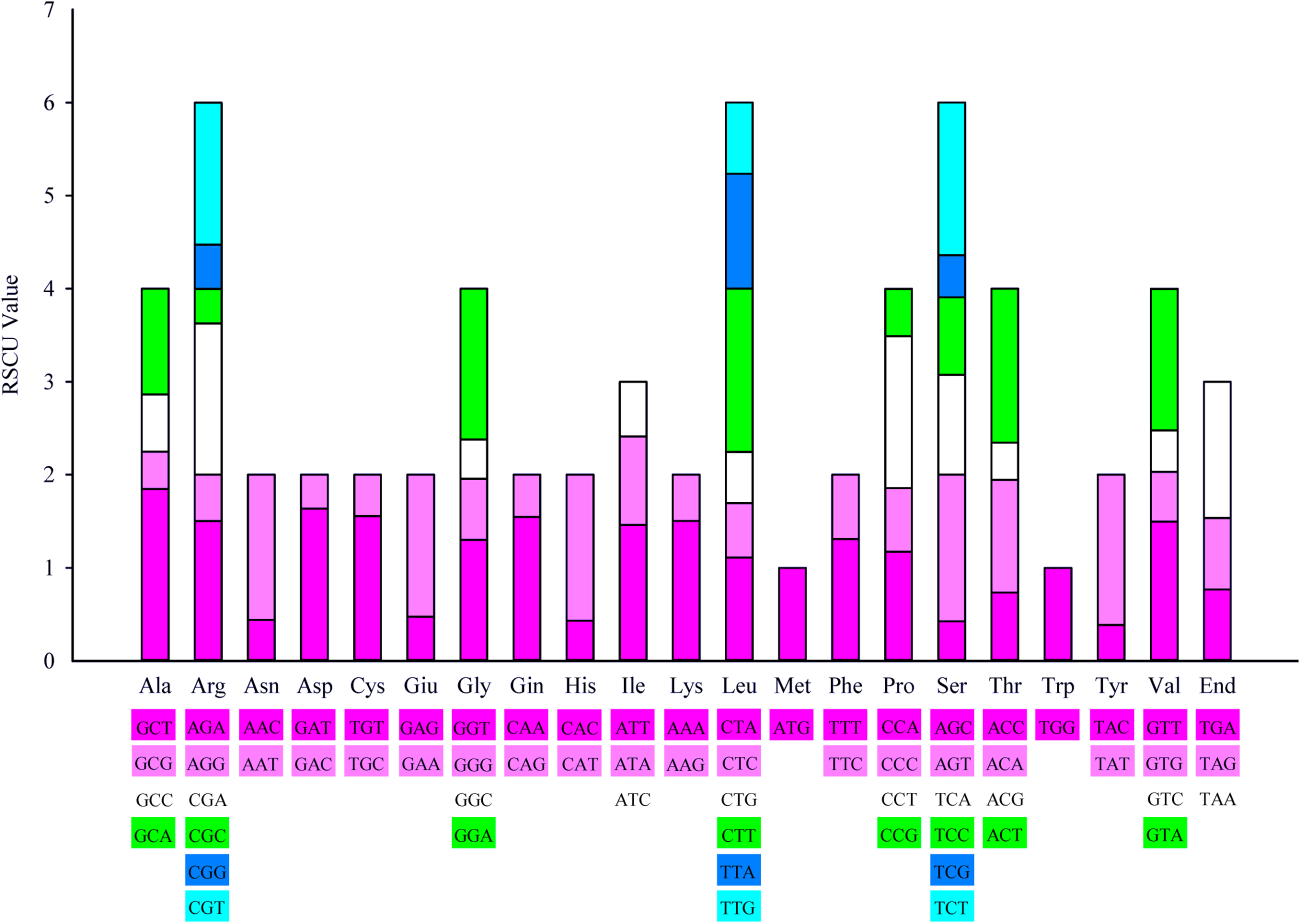
20 amino acid codon and stop codon of the island plant Hainan *C. oleifera* chloroplast genome. The color of the histogram corresponds to the color of the codon.

In the Hainan *C. oleifera* chloroplast DNA, the 88 protein-coding genes had 27,270 codons. More than 94% of the protein-coding genes used ATG as the start codon, and less than 6% of the remaining used ATT (*petB* and *rps19*), ATA (*ndhD*), and ATC (*rpl16* and *orf42*) as start codons. Furthermore, ATA, ATC, ATT, and TTG were used as start codons in the *Aquilaria sinensis* chloroplast DNA (Wang et al., 2016), and GTG and ACT served as start codons in the *rps19* and *rps2* genes in *Morus atropurpurea*, respectively (Li et al., 2016). Among the stop codons, TAA was the most common, followed by TAG and TGA, with 73.86% of the stop codons ending in A, thus strongly reflecting the AT bias in codon usage. The RSCU value of the Hainan *C. oleifera* chloroplast genome was shown in Fig. 5; its value increased with the number of codons. Leucine accounted for the highest codon usage (10.84%), followed by serine (9.17%) and isoleucine (8.42%). Nearly one third of the total codons were represented by these three amino acids.

**Figure 6:**
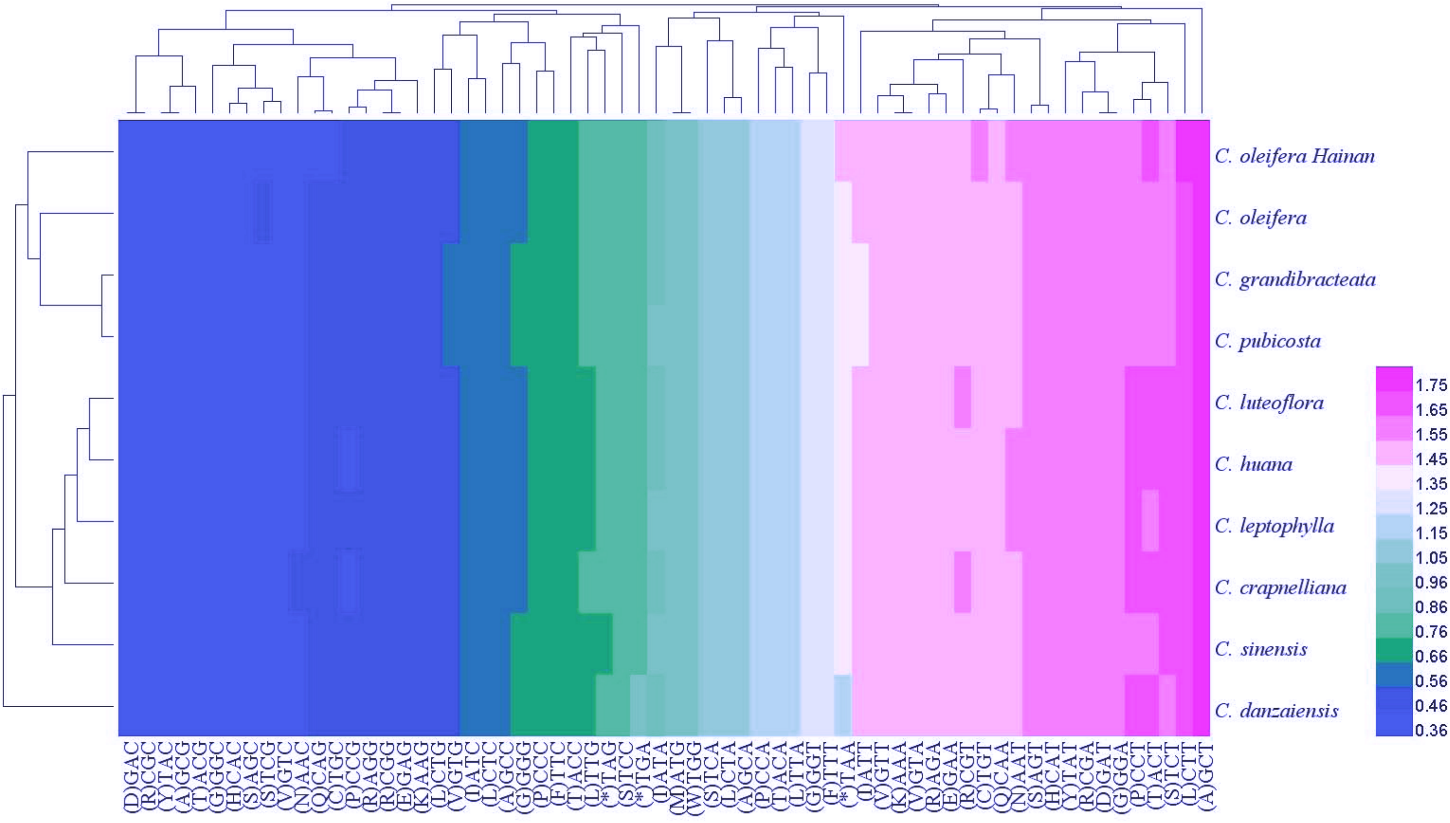
The distributions of codon usage in the form of heat maps for 10 Theaceae species. Color indication: red represents the larger RSCU values and blue represents the smaller RSCU values.

The distributions of codon usage in the form of heatmaps for 10 Theaceae species were shown in Fig. 6. It can be seen that about half of the codons with low RSCU values (shown in blue in the heatmaps) were infrequently used. All codons with RSCU >1 ended with A/T. A similar phenomenon has been found in other plant lineages, indicating that the A + T bias plays an important role in the plastid genome. In addition, we found that the codon usage of Hainan *C. oleifera* chloroplast genome was most similar to that of *C. oleifera*. As more chloroplast genome data are being continually explored, further validation of these findings will allow us to understand the relationship between different codon usage patterns and hosts, as well as the interactions among nuclear genomes (Liu & Xue, 2005).

### Phylogenetic relationship with near source species

**Figure 7:**
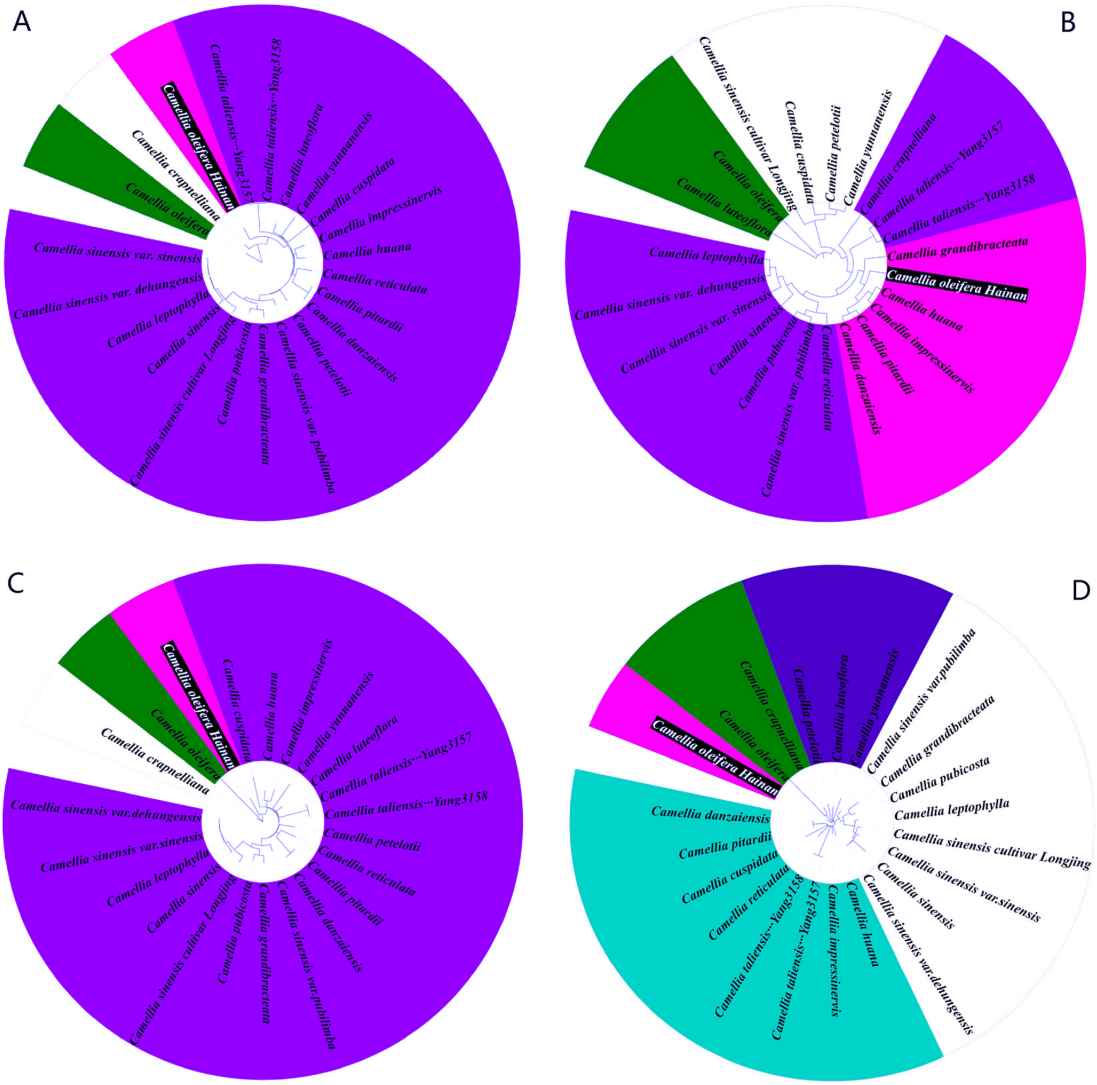
Phylogenetic relationships of 22 species of *Camellia* plants inferred from different data partitions. (A) Whole chloroplast genome; (B) protein coding region; (C) LSC region; (D) SSC region Species with similar evolutionary relationships are displayed in the same color.

As an important part of plant cell organelles and photosynthetic organs, chloroplasts play an important role in the long history of biological evolution (Kim & Archibald, 2009). In this study, we not only constructed the phylogenetic tree of complete chloroplast genome of 22 *Camellia* species, but also extracted protein sequence information, LSC region sequences and SSC region sequences, and constructed the phylogenetic tree. The results showed a different evolutionary relationship, in which the whole chloroplast genome showed that Hainan *C. oleifera*, *C. oleifera*, and *C. crapnelliana* were independent branches and have similar evolutionary relationships (Fig. 7A). The protein coding sequence showed that Hainan *C. oleifera* was closely related to *C. crapnelliana*, *C. danzaiensis*, *C. pitardii*, *C. huana*, and *C. impressinervis*, which was closest to *C. crapnelliana* (Fig. 7B). The results of the LSC region (Fig. 7C) and the SSC region (Fig. 7D) were similar to those of the complete chloroplast genome sequence, showing that the three Camellia species of Hainan *C. oleifera*, *C. oleifera*, and *C. crapnelliana* were the same family. The evolutionary relationship was clear. There were similar parts in these results, especially *C. oleifera* and *C. crapnelliana*, but the protein coding sequence is more stable and not susceptible to the external environment, so its phylogenetic relationship is more informative.

The base sequence comparison of DNA barcodes (*rbcL*, *matK*, and *trnH-psbA*) revealed a high degree of conservation of the chloroplast genome sequence of the *Camellia* plants (Table S3). There were 10 locus in *rbcL* with differences (marked by red triangles below), but Hainan *C. oleifera* has the same base composition as most species. There were 22 locus differences in *matK*, similar to *rbcL*, the most occurred in a single base region of other species. Unlike the other species, Hainan *C. oleifera* was replaced by T at one base, and the rest were C. In the *psbA-trnH* comparison, there were nine positions in which the base changes slightly, while in the second change point, Hainan *C. oleifera* was C, and other species were A. Therefore, these single site changes can be used as good molecular markers.

## Discussion

With the rapid development of molecular biology techniques, new molecular markers and detection methods are emerging in an endless stream. Several DNA analysis techniques suitable for chloroplast genome research on plant phylogeny and evolution will be available. Moreover, the research on population genetic changes will play an important role in the protection of biological resources and biodiversity (Xu & Wang, 2001). As an important evolutionary feature of the chloroplast genome, the codon usage bias has become an analytical tool widely used in several organisms (Nie et al., 2014; Sharp et al., 1988; Vicario, Moriyama & Powell, 2007). In this study, we systematically analyzed the RSCU value of the Hainan *C. oleifera* chloroplast genome. The results showed that nearly half of the values were higher than 1.00 and nearly half were lower than 1.00, and all high-valued codons ended with A or T, whereas the RSCU value was 1.00 without any codon usage bias (Gupta, Bhattacharyya & Ghosh, 2004). Therefore, we hypothesized that the codons in Hainan *C. oleifera* chloroplast genome preferably ended with A/T. This conjecture was also confirmed in this paper, and has also been found in other plant studies (Wang et al., 2016). It is generally accepted that codon usage bias reflects the balance between the mutational bias and natural selection for translation optimization (Bulmer, 1988; Sharp et al., 1993; Shin et al., 2015). In order to further clarify the role of the two in the evolution of the Asteraceae species, some researchers used the neutrality analysis, which reflected that the natural selection pressure played a major role in shaping codon usage (Nie et al., 2014). However, the *C. oleifera* population on Hainan Island has a certain geographical isolation; the genetic variation of population might occur. Whether the mutation or natural selection plays a leading role needs further confirmation.

Previous studies have shown that the chloroplast genome structure is highly conserved (Korpelainen, 2004). In this study, the genomic composition, gene sequence, GC content, and codon preference of the Hainan *C. oleifera* chloroplast genome were not much different from that of the other *Camellia* species, and were similar to the typical angiosperm chloroplast genome (Shinozaki et al., 1986; Wang et al., 2016; Yang et al., 2013), which revealed that the chloroplast genome of Hainan *C. oleifera* is highly conserved in structure and evolution. The conservation of these sequences makes Hainan *C. oleifera* have some pressure on evolutionary research, so that it difficult to determine the differential genes it contains. This also forced research on the evolution of *C. oleifera* on Hainan Island. In addition, we focused on the junctions between the IR/LSC and IR/SSC regions of the chloroplast genome, where more sequence data were available. In the long process of evolution, the structural order of the LSC, SSC, and IR regions remained unchanged. The differences among the chloroplast genomes of different species are mainly reflected in the length and orientation of the IRs regions. Among them, a similar situation was produced in Hainan *C. oleifera*, *C. crapnelliana*, and *C. danzaiensis* due to the change of IRa region, and the similarity at this level indicated that they shared a common ancestor. This can initially determine its source to reduce the pressure of its taxonomic research. As an important indicator of chloroplast genome evolution, the contractions, and expansions of the IR/LSC and IR/SSC boundaries often determine the chloroplast genome length variation (Goulding et al., 1996; Zhang et al., 2013). Moreover, the boundaries of the plastids of various species are often different even between members of the same family (Ravi et al., 2006). Therefore, it would be useful to compare the IR/LSC and IR/SSC junctions of chloroplast genome in different *Camellia* species for alleviating its evolutionary pressure.

It was difficult to study the classification and phylogeny of the genus *Camellia* because of various reasons, such as its frequent interspecific hybridization and polyploidization (Huang et al., 2014; Yang et al., 2013). However, the samples of *C. oleifera* used in this study were grown in a unique island environment, and their genetics and evolutionary history are still vague. Even if we used the complete chloroplast genome information for analysis, it was not enough to completely solve all phylogenetic relationships, as can be seen in previous studies (Petersen et al., 2011; Steane, 2005; Wortley et al., 2005). Besides, the comparison object we selected were no plant groups involved in the other genera of the family Theaceae, which might provide useful information for the evolutionary study of Hainan *C. oleifera*. In summary, our research can promote the exchange of information between the nuclear genomes of *Camellia* species and provide valuable genomic resources for phylogenetic studies.

## Conclusions

In this study, we sequenced the complete chloroplast genome of *C. oleifera* on Hainan Island, China, using the Illumina high-throughput sequencing technology. The chloroplast genome of Hainan *C. oleifera* was found to have an intact quadripartite structure by genome sequencing and reassembly. The annotation and comparison with other *Camellia* species showed that the genomic composition, gene sequence, GC content, and codon preference of the Hainan *C. oleifera* chloroplast genome were not much different from that of the other *Camellia* species, and were similar to the typical angiosperm chloroplast genome. Therefore, we performed in-depth analysis of the border regions, long repeats, SSRs, and sequence diversity in the chloroplast genomes of 10 *Camellia* plants, indicated that their chloroplast genomes were highly conserved in structure and evolution. Long-sequence repeats, SSRs and DNA barcodes can be used as novel molecular markers. By studying the gene composition of the marginal regions, codon distribution and phylogeny, it was found that Hainan *C. oleifera* has similar evolutionary relationships with *C. crapnelliana*. Therefore, the data obtained in this study will be helpful to further study the evolutionary history of Hainan *C. oleifera* and to protect its excellent germplasm resources, and to provide valuable genome resources for phylogenetic and taxonomic studies and for the exchange of information between nuclear genomes.

## Supplemental Information

10.7717/peerj.7210/supp-1 Supplemental Information 1 The genes with introns in the Hainan *C. oleifera* chloroplast genome and the lengths of introns and exons. ^*^The *ycf1* is a trans-spliced gene with the start located in the SSC region and the end in the IRb region, and possibly missing 3’ end.

10.7717/peerj.7210/supp-2 Supplemental Information 2 Distribution of simple sequence repeats (SSRs) in 10 Theaceae chloroplast genomes.

10.7717/peerj.7210/supp-3 Supplemental Information 3 Comparison of base changes in DNA barcodes (rbcL, matK and trnH-psbA).

## Additional Information and Declarations

### DNA Deposition

The following information was supplied regarding the deposition of DNA sequences: The complete chloroplast genome sequence of *C. oleifera* is available at GenBank: MF541730.

### Data Availability

The following information was supplied regarding data availability: Data access in NCBI: all complete chloroplast genome sequence are available with the following accessions Hainan *C. oleifera* (MF541730), *C. oleifera* (JQ975031), *C. huana* (KY626040), *C. impressinervis* (KF156835), *C. pitardii* (KF156837), *C. danzaiensis* (KF156834), *C. grandibracteata* (KJ806274), *C. leptophylla* (KJ806275), *C. sinensis var. sinensis* (KJ806281), *C. sinensis var. dehungensis* (KJ806279), *C. crapnelliana* (KF753632), *C. taliensis* voucher HKAS S.X.Yang3157 (KF156839), *C. taliensis* voucher HKAS S.X.Yang3158 (KF156836), *C. reticulata* (KJ806278), *C. sinensis var. pubilimba* (KJ806280), *C. pubicosta* (KJ806277), *C. sinensis* (KY626042), *C. sinensis* cultivar Longjing (KF562708), *C. cuspidata* (KF156833), *C. yunnanensis* (KF156838), *C. petelotii* (KJ806276), *C. luteoflora* (KY626042). The authors state that all data necessary for confirming the conclusions presented in the article are represented fully within the article.
